# Lesions of the Lateral Habenula Increase Voluntary Ethanol Consumption and Operant Self-Administration, Block Yohimbine-Induced Reinstatement of Ethanol Seeking, and Attenuate Ethanol-Induced Conditioned Taste Aversion

**DOI:** 10.1371/journal.pone.0092701

**Published:** 2014-04-02

**Authors:** Andrew K. Haack, Chandni Sheth, Andrea L. Schwager, Michael S. Sinclair, Shashank Tandon, Sharif A. Taha, for the Brain Institute

**Affiliations:** 1 Department of Neurobiology and Anatomy, University of Utah School of Medicine, Salt Lake City, Utah, United States of America; 2 Interdepartmental Program in Neuroscience, University of Utah School of Medicine, Salt Lake City, Utah, United States of America; 3 Department of Pharmacology and Toxicology, University of Utah School of Medicine, United States Salt Lake City, Utah, United States of America; Radboud University, Netherlands

## Abstract

The lateral habenula (LHb) plays an important role in learning driven by negative outcomes. Many drugs of abuse, including ethanol, have dose-dependent aversive effects that act to limit intake of the drug. However, the role of the LHb in regulating ethanol intake is unknown. In the present study, we compared voluntary ethanol consumption and self-administration, yohimbine-induced reinstatement of ethanol seeking, and ethanol-induced conditioned taste aversion in rats with sham or LHb lesions. In rats given home cage access to 20% ethanol in an intermittent access two bottle choice paradigm, lesioned animals escalated their voluntary ethanol consumption more rapidly than sham-lesioned control animals and maintained higher stable rates of voluntary ethanol intake. Similarly, lesioned animals exhibited higher rates of responding for ethanol in operant self-administration sessions. In addition, LHb lesion blocked yohimbine-induced reinstatement of ethanol seeking after extinction. Finally, LHb lesion significantly attenuated an ethanol-induced conditioned taste aversion. Our results demonstrate an important role for the LHb in multiple facets of ethanol-directed behavior, and further suggest that the LHb may contribute to ethanol-directed behaviors by mediating learning driven by the aversive effects of the drug.

## Introduction

The lateral habenula (LHb) has been importantly implicated in learning driven by adverse outcomes. Neurons in the primate LHb are excited by negative stimuli, such as an aversive air puff or cues predicting the absence of reward [1], [2]. Excitation of LHb neurons results in inhibition of dopamine neurons in the ventral tegmental area and substantia nigra pars compacta [3]. This inhibition is mediated by a disynaptic pathway in which excitatory afferents originating in the LHb synapse on GABAergic neurons in the rostromedial tegmental area (RMTg), which target and inhibit downstream dopaminergic neurons [4]. Supporting a role for the LHb-RMTg pathway in learning driven by undesirable outcomes, manipulations that increase firing in the LHb, in LHb efferents to the RMTg, or in the RMTg itself are both acutely aversive and cause aversive conditioning [5]–[11].

The positively reinforcing effects of drugs of abuse motivate further drug seeking, particularly in initial stages of drug use [12]. However, these drugs also have aversive effects that limit voluntary intake [13]. Recent studies have implicated the habenula in negatively regulating motivation for both nicotine [14] and cocaine [6], [15], and provide evidence that habenular circuits mediate learning driven by the aversive effects of these drugs. Ethanol consumption results in aversive effects that include acute sedation and motoric impairment as well as delayed hangover effects [16], [17]. Sensitivity to these aversive effects is associated with decreased voluntary ethanol intake in rodent models, as shown by the inverse correlation of ethanol-induced conditioned taste aversion (CTA) with voluntary ethanol consumption and preference [18], [19]. Along these lines, increased ethanol intake in adolescent rats (compared to adults) is accompanied by decreased ethanol CTA [20]; moreover, CTA magnitude in individual adolescent rats is inversely related to subsequent voluntary ethanol intake [16]. Together these results suggest an important role for the aversive effects of the drug in suppressing voluntary ethanol consumption. Importantly, these effects are likely to be clinically relevant, as decreased sensitivity to the effects of ethanol, including aversive effects, is predictive of higher levels of binge drinking and higher risk for development of an alcohol use disorder in human populations [21]–[23].

The neural circuitry through which the aversive effects of ethanol suppress voluntary consumption is not well defined. Evidence supporting a role for the LHb in learning driven by aversive outcomes, including those caused by other drugs of abuse, raises the possibility that this brain region could contribute to these suppressive effects. To begin characterizing the role of the LHb in ethanol-directed behaviors, we studied voluntary ethanol consumption, ethanol self-administration, yohimbine-induced reinstatement of ethanol seeking, and ethanol-induced CTA in LHb and sham-lesioned rats. Our results show that lesions of the LHb increase both voluntary ethanol intake and self-administration, block yohimbine-induced reinstatement of ethanol seeking, and attenuate a taste aversion conditioned by a single noncontingent ethanol injection.

## Materials and Methods

### Ethics statement

All procedures used were approved by the University of Utah Animal Care and Use Committee and carried out in accordance with the National Institutes of Health Guide for the Care and Use of Laboratory Animals.

### Subjects

136 male Long-Evans rats (300–350 g at the time of receipt; Charles-River, Wilmington, MA,) were used in the present experiments. Rats were single-housed in Plexiglas tub cages and maintained on a 12 hour light/dark cycle. *Ad libitum* access to food and water was available throughout all experimental procedures. A summary of the experimental groups and the timeline of experimental procedures (described below) within each group is provided in Table 1.

**Table 1:** Summary of experimental groups and timeline of procedures. Numbered experiments indicated the order in which experiments within each group were carried out.

Rat group	Number of rats		Experiments
	Sham	Lesioned	
1	7	7	1) Intermittent ethanol access
			2) 24 h timeline of ethanol intake
			3) Effects of abstinence on IEA intake
			4) Taste preference
2	10	10*	1) Intermittent ethanol access
			2) Operant ethanol self-administration, extinction, and reinstatement
3	7	8	1) Operant sucrose self-administration, extinction, and reinstatement
4	37	42	1) Ethanol conditioned taste aversion
5	4	4	1) Ethanol metabolism

* A single lesioned rat died during intermittent ethanol access; the 9 remaining lesioned rats were trained in operant ethanol self-administration.

### Drugs

Ethanol (Decon Labs, King of Prussia, PA) solutions were prepared in filtered tap water to a concentration of 20% (v/v) for use in the intermittent ethanol access paradigm, and prepared in physiological saline to a concentration of 20% for the CTA experiment. Saccharin, quinine and yohimbine (Sigma Aldrich, St. Louis, MO) solutions were prepared in distilled water.

### Electrolytic lesion of the LHb

Surgical anesthesia was induced and maintained with isoflurane (5% and 2%, respectively). The skull was exposed and burr holes drilled bilaterally above the LHb. Lesions were produced by passing current (0.5 mA, 10 s) through a stainless steel electrode (AM Systems, Sequim, WA) at two sites within each hemisphere that targeted anterior and posterior portions of the LHb. Coordinates for these sites (in mm from bregma) were: –3 and –3.7 posterior; 0.7 lateral; and –5.4 ventral. In sham-lesioned animals, electrodes were lowered to stereotaxic coordinates 1 mm dorsal to the LHb but no current was passed at the target site. Rats were allowed to recover for at least one week after surgery before experiments commenced.

### Voluntary ethanol consumption

Voluntary ethanol consumption was measured using an intermittent ethanol access (IEA) two bottle choice paradigm [24]–[26]. Rats (n = 34; 17 sham and 17 lesioned) were given 24 hour access to 20% (v/v) ethanol and water in a two bottle choice paradigm in the home cage 3 times per week (Monday, Wednesday and Friday). Ethanol bottles were placed in home cages at 9 a.m. on access days. Ethanol and water bottle positions within each cage were alternated in successive drinking sessions to minimize the effect of side preferences. On days in which ethanol was not presented, *ad lib* water was available. IEA was provided for a minimum of 8 weeks (24 drinking sessions). Ethanol and water intake were measured by weighing bottles before and after each 24 hour access period. These measures were used to calculate ethanol intake (normalized to weight, g/kg/24 h) and preference (ethanol intake/total fluid intake). Body weight and food intake were measured weekly during IEA.

In a subset of sham and lesioned rats (n = 7 rats in each group; see Table 1), the timeline of ethanol intake over 24 hour access periods was investigated by measuring intake in the first hour of ethanol access (9–10 am), the remainder of the light cycle (10 am – 6 pm), the first hour of the dark cycle (6–7 pm), and the remainder of the 24 hour period (7 pm – 9 am). These measurements were carried out in rats that had IEA for 9 weeks (27 drinking sessions). In the same group of rats, the stability of ethanol intake after a period of abstinence was investigated. After an initial period of 10 weeks of IEA, rats were subjected to nearly 7 weeks (46 days) without ethanol access. IEA was then restored for two additional weeks to allow comparison of ethanol intake before and after abstinence.

### Taste preference: two-bottle choice for saccharin and quinine solutions

Preference for saccharin solutions (0.005, 0.01, 0.05, 0.1, 0.5, 1 and 5 mM concentrations) and aversion to quinine solutions (0.0003, 0.001, 0.003, 0.01, 0.03, 0.1, 0.3 mM) were assessed using a two-bottle choice paradigm in the home cage in a subset of sham and lesioned rats (n = 7 rats in each group). In this paradigm, a single bottle of tastant was made available for 48 hours in the home cage, concurrent with a single bottle of water; intake of the tastant and water were measured every 24 hours, and bottle positions were switched after the first 24 period. Intake during the two 24 hour periods was averaged to produce a single measure of consumption for each tastant concentration. Tastants were presented in sequential order of increasing concentration in consecutive 48 hour intervals. Intake during access to the quinine concentration series was measured first, followed by intake during presentation of the saccharin concentration series. To investigate the timeline of saccharin consumption over 24 hour access periods, intake of highly preferred 5 mM saccharin was measured during the four intervals in which ethanol intake was measured: 9–10 am, 10 am–6 pm, 6–7 pm, and 7 pm–9 am.

### Operant responding for ethanol

After a minimum of two months home cage access to 20% ethanol in the IEA paradigm, rats (n = 10 sham and 9 lesioned rats) were trained to self-administer 20% ethanol. Operant chambers (Med Associates, St. Albans, VT) were equipped with a central reward receptacle flanked by retractable levers, with illuminated cue lights over each lever. Operant chambers were enclosed in sound attenuating chambers equipped with fans that increased ventilation and provided masking noise. Ethanol was delivered to the reward receptacle via a programmable syringe pump.

In an initial overnight session, rats were trained on an FR1 schedule to respond for 20% ethanol. In this overnight session, only the active lever was presented to facilitate learning. Each lever press caused the active lever to retract, the associated cue light to extinguish, and resulted in immediate delivery of 0.1 mL 20% ethanol into the reward receptacle. After 5 seconds, the active lever was extended and the cue light illuminated. After the initial overnight session, rats were trained in 1 hour sessions three times per week (Monday, Wednesday, Friday) using an FR1 schedule in which only the active lever was available. After 2 weeks, rats progressed to an FR3 schedule in which the session length was decreased to 30 minutes and the inactive lever was introduced. Lever presses on the inactive lever were recorded but had no programmed consequences. Responses in this paradigm were measured over two weeks before extinction training began (below).

### Extinction and reinstatement of ethanol seeking

During extinction sessions, the syringe containing 20% ethanol was removed from the syringe pump, and thus active lever presses no longer resulted in ethanol delivery. In all other respects, the extinction paradigm was identical to the final operant response paradigm described above, including presentation of visual and auditory cues (cue light extinguishment, lever retraction, and syringe pump activation). Extinction sessions were run on alternate weekdays for four successive sessions before testing for yohimbine-induced reinstatement.

Reinstatement was studied by administration of the α2 receptor antagonist yohimbine (2 mg/kg, IP) or vehicle solution (distilled water) 30 minutes prior to testing in extinction sessions. Because yohimbine reliably induces multiple reinstatements of ethanol seeking [27], reinstatement in each animal was measured after each of two yohimbine injections to minimize variability in behavioral results. During reinstatement testing, rats were first tested for operant responding in an extinction session after injection of the vehicle solution. This was followed by a rest day (no injection or operant session). The next day, reinstatement of ethanol seeking after yohimbine injection was tested. Two additional extinction sessions followed, and then an identical testing schedule (vehicle injection, rest day, yohimbine injection) was carried out. For each animal, responses were averaged across each of the two drug administrations to yield a single measure of operant responding after vehicle administration and after yohimbine administration.

### Sucrose: Operant responding, extinction and reinstatement

Fifteen ethanol-naive rats (7 sham and 8 lesioned rats) were trained to operantly self-administer 2% sucrose. With the exception of the use of sucrose as a reinforcer, the paradigm used was identical to the final operant paradigm used in ethanol self-administration (30 minute FR3 task with both active and inactive levers available). Training in this group was similar to that described for ethanol self-administration above, and began with an initial overnight training session (FR1, no inactive lever). In five subsequent sessions, the response requirement was maintained at FR1 but the session duration was shortened to a single hour. Finally, rats in this group progressed to the final paradigm (FR3, half hour duration, inactive lever introduced) for ten successive sessions.

Thereafter, extinction followed by reinstatement sessions were carried out. The extinction paradigm was identical to that described for extinction of ethanol seeking. A total of seven successive extinction sessions were run in this experiment. This increase (over four sessions used in extinction of ethanol seeking) was incorporated because response rates for sucrose self-administration were substantially higher than those occurring during ethanol self-administration. Yohimbine-induced reinstatement was tested in this experiment in a manner identical to that described above in studies of ethanol reinstatement.

### Conditioned taste aversion

A total of 79 ethanol-naïve animals (37 sham and 42 lesioned male Long-Evans rats) were included in the CTA experiment. After lesion surgery, all rats were single housed and allowed at least one week recovery in home cages. Thereafter, rats were habituated to handling for two days before beginning the CTA experiment. Throughout the experiment, rats were maintained with *ad lib* access to both food and water. We avoided water deprivation often used in CTA paradigms because dehydration causes anorexia [28]. Food deprivation has been shown activate LHb neurons [29], [30], raising the concern that water deprivation and associated dehydration-induced anorexia might differentially affect motivation in sham and LHb-lesioned rats. To induce consumption, a highly palatable supersaccharin solution (0.125% saccharin + 3% glucose) was used as the conditioned stimulus.

The first day of the CTA experiment, rats were given 30 minutes home cage access to supersaccharin. Rats were then assigned to saline or ethanol injection groups, with mean supersaccharin consumption matched between groups. Rats in the resulting four groups (LHb lesion/sham x saline/ethanol injection) were immediately injected with either ethanol (0.7 g/kg of 20% ethanol, IP) or saline (volume matched to ethanol injections) and returned to their home cages. The ethanol dose used was based on pilot studies showing it produced reliable but not saturating CTA. On each of the next six days, rats again received a single period of 30 minutes home cage access to the supersaccharin solution and consumption was measured. Supersaccharin access sessions, including the initial session, occurred between 4 and 6 pm daily.

### Measurement of blood ethanol concentration

Blood ethanol concentration (BEC) was measured after voluntary ethanol intake in the IEA paradigm and operant self-administration sessions. BECs after ethanol consumption in the IEA paradigm were measured from tail vein blood collected after the first 30 minutes of ethanol access, the interval during which previous results [31] and our own measurements suggested intake rates were highest. BECs were measured in rats that had received 14 weeks of IEA. BECs after operant responding for ethanol were measured in tail vein blood collected immediately after 30 minute self-administration sessions.

To determine if LHb lesion altered ethanol metabolism, we measured BECs after noncontingent injection of ethanol (1 g/kg 20% ethanol, IP) in a separate group of ethanol-naïve rats (4 sham and 4 lesioned).

Blood samples were collected into heparinized capillary tubes at 15, 30, 60, 120 and 180 minutes after injection. For all BEC measurements, blood plasma was isolated from samples by perchloric acid precipitation and brief centrifugation (2000 rpm, 5 m). BEC was measured using the NAD-NADH enzyme spectrophotometric method [32], [33].

### Analysis of lesions

Rats were deeply anesthetized with pentobarbital and perfused with physiological saline followed by 4% formaldehyde. Brains were cryoprotected and sectioned in 50 µm slices. Sections were mounted and Nissl stained. Damage to the LHb was localized by comparison to a reference atlas [34] by an observer blind to behavioral results. Two lesioned rats died during or shortly after experimental procedures and lesion sites were not analyzed in these animals.

Though damage was largely confined to the LHb, some lesion sites encroached upon surrounding structures, including the medial habenula (MHb). To determine if damage to this structure contributed to voluntary ethanol intake in the IEA paradigm, we quantified the extent of this damage in each lesioned animal. The damage to the MHb in each hemisphere was estimated by visual inspection, and was scored as 0% (no damage), 25%, 50%, 75%, or 100% (complete ablation). Scores for each hemisphere were averaged to produce a single estimate of damage to the MHb within each rat. Rats were divided into high and low damage groups by performing a median split of this data, and ethanol intake in the IEA paradigm was then compared between groups.

### Statistical analysis

Voluntary ethanol intake, escalation of ethanol intake, taste preference, and reinstatement of ethanol and sucrose seeking were analyzed using two-way repeated measures ANOVA. All analyses included lesion (sham or LHb) as one factor. The second factor consisted of IEA drinking session (voluntary ethanol intake); time interval (escalation of intake); tastant concentration (taste preference); or drug (reinstatement experiments; yohimbine or vehicle treatment). Extinction of ethanol-self administration and ethanol-induced CTA were analyzed using two- (factors of lesion and time) and three-way (factors of lesion, drug and time) ANCOVA, respectively. Baseline ethanol self-administration and supersaccharin intake were used as covariates in these analyses, respectively. Where appropriate, Holm-Sidak posthoc tests were used. BECs and operant self-administration were analyzed using Pearson’s correlation test and t-tests.

## Results

### Voluntary ethanol consumption

**Figure 1:**
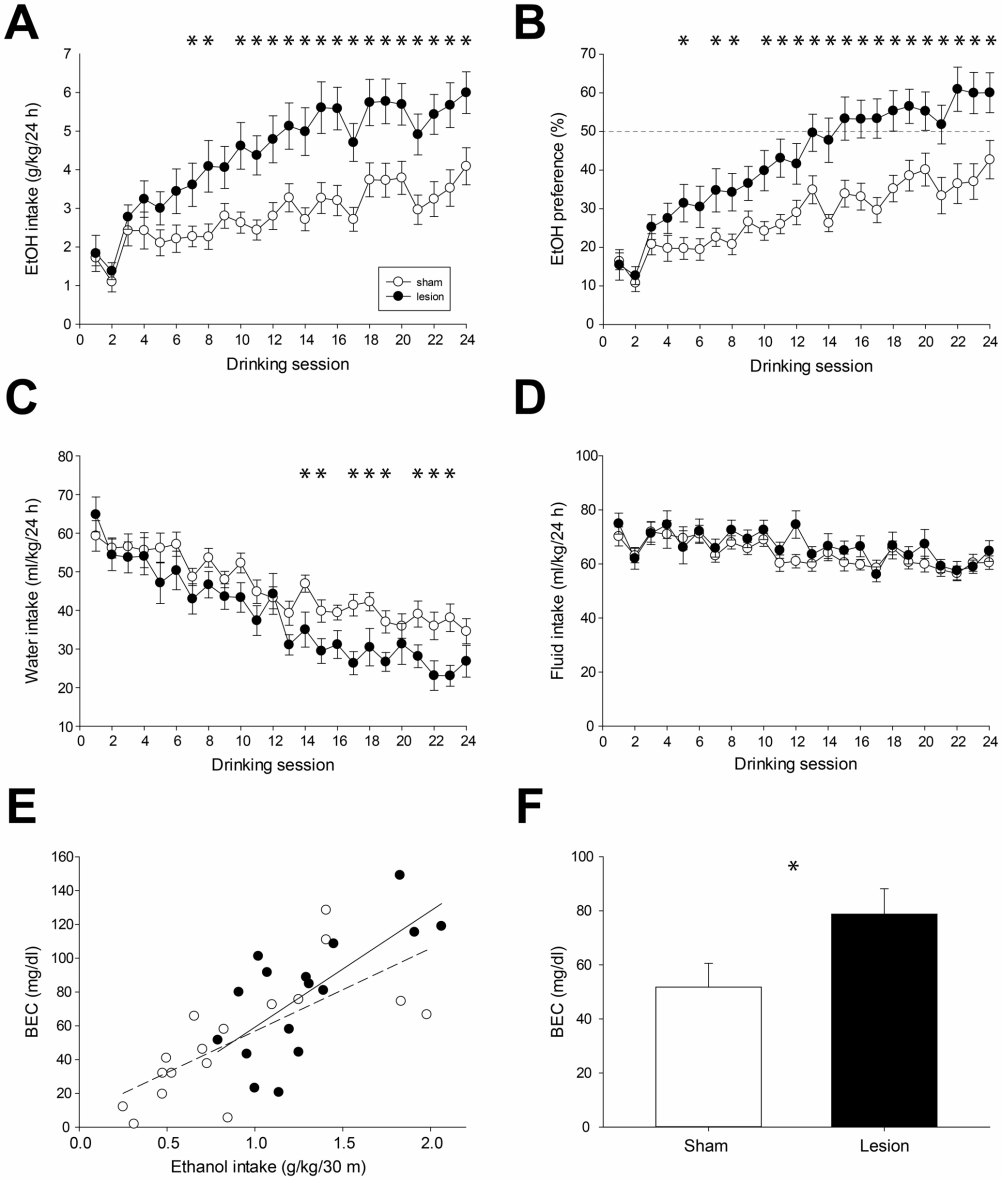
Voluntary ethanol consumption during intermittent access. (A) Lesioned rats consumed significantly more 20% ethanol over the course of 2-bottle choice drinking sessions (3 sessions/week for 8 weeks, 24 sessions total). Symbols indicate mean ethanol intake ± SEM. In this figure and Figures 2–5, filled symbols indicate lesioned animals, open symbols indicate sham animals, and asterisks indicate significant differences (p<0.05). (B) Alcohol preference in lesioned rats was significantly higher than that in sham rats. (C) Water intake decreased progressively in both groups, and rats in the lesioned group consumed significantly less water starting in the 5^th^ week (14^th^ session) of the paradigm. (D) Total fluid intake (water + ethanol) did not differ significantly between the two groups. (E) Blood ethanol concentrations (BECs) were significantly correlated with voluntary ethanol intake for both groups. Broken line indicates linear fit for sham group; solid line indicates linear fit for lesioned group. (F) Mean (± SEM) BEC measured after the first 30 minutes of 20% ethanol access was significantly higher in lesioned vs. sham rats (p<0.05).

Intermittent home cage access to 20% ethanol (IEA) in a two bottle choice paradigm resulted in steady escalation of ethanol consumption in both sham and lesioned animals (Fig. 1A; main effect of drinking session, F(23, 728)  = 28.2, p<0.001). However, intake in the lesioned group diverged from that in the sham group after the first week (drinking sessions 1–3), escalated more rapidly over the course of the next several weeks, and plateaued at higher stable levels of ethanol intake (main effect of lesion, F(1, 32)  = 9.3, p<0.01; significant interaction of lesion x drinking session, F(23, 728)  = 3.6, p<0.001). Differences in ethanol consumption between sham and lesioned animals reached significance during the seventh drinking session and remained significant from the tenth through the final drinking session (p<0.05, posthoc tests). Ethanol consumption in the final drinking session averaged 4.1±0.5 and 6.0±0.5 g/kg/24 h for sham and lesioned animals, respectively. Differences in these normalized measures of ethanol intake were due solely to different levels of ethanol consumption, as mean rat weight did not differ between the two groups during IEA (data not shown; no significant effect of lesion on body weight, F(1, 32)  = 0.6, NS; and no significant interaction of lesion x time, F(7, 224)  = 0.7, NS; also no significant effect of lesion on food intake F(1, 8)  = 0.0, NS; and no significant interaction of lesion x time, F(8, 96)  = 0.9, NS). Similar differences were apparent in measures of ethanol preference (Fig. 1B), which averaged 43±5% and 60±5% in sham and lesioned animals, respectively, in the final drinking session (main effect of session, F(23, 728)  = 32.2, p<0.001; main effect of lesion, F(1, 32)  = 11.0, p<0.01; significant interaction of lesion x session, F(23, 728)  = 2.9, p<0.001). Posthoc testing indicated that differences between sham and lesioned rats were significant in drinking sessions 5, 7–8, and 10–24 (p<0.05, posthoc tests).

Water consumption in both groups decreased progressively over the course of drinking sessions (Fig. 1C; main effect of session, F(23, 736)  = 26.7, p<0.001). Intake in lesioned rats was significantly lower than that in sham rats (main effect of lesion, F(1, 32)  = 5.1, p<0.05; significant interaction of lesion x session, F(23, 736)  = 1.7, p<0.05). Differences in water intake reached significance first in the 14^th^ drinking session and were intermittently significant thereafter (p<0.05, posthoc tests). Total fluid intake (water plus ethanol consumption) did not differ between the two groups (Fig. 1D; no significant effect of lesion, F(1, 32)  = 0.62, NS; no significant interaction of lesion x session, F(23, 736)  = 1.2, NS).

To determine if differences in voluntary ethanol consumption resulted in different blood ethanol concentrations (BECs), we measured ethanol concentration in tail vein blood collected after the first 30 minutes of ethanol access in the IEA paradigm. BEC measures were significantly correlated with ethanol consumption occurring during this period for both sham and lesioned rats (Fig. 1E; r^2^ = 0.49 and 0.52 for lesioned and sham rats respectively, p<0.01 for each group). Lesioned rats consumed more ethanol during this period (mean ± SEM of 1.3±0.1 vs. 0.9±0.1 g/kg, p<0.05) and reached significantly higher BECs (Fig 1F; 79±9 vs. 52±9 mg/dl, p<0.05).

To determine if LHb lesion altered ethanol metabolism, we measured BECs at various time points after a single ethanol injection (1 g/kg 20% ethanol, IP; BEC measured 15, 30, 60, 120, and 180 m after injection) in a separate group of previously ethanol-naïve rats (n = 4 sham and 4 lesioned). BECs in these groups did not differ at any time point after ethanol injection (Table 2; no effect of lesion, F(1, 6) = 0.04, NS; no interaction of time x lesion, F(6, 24)  = 0.9, NS), suggesting that lesion of the LHb had no effect on ethanol metabolism.

**Table 2:** BECs (mg/dl) in ethanol naïve sham and lesioned rats following 1 g/kg IP ethanol injection.

	Time (minutes) after ethanol injection				
	15	30	60	120	180
Sham	129±15	118±7	108±9	76±11	31±11
Lesioned	118±18	108±14	109±11	77±4	38±9

**Figure 2:**
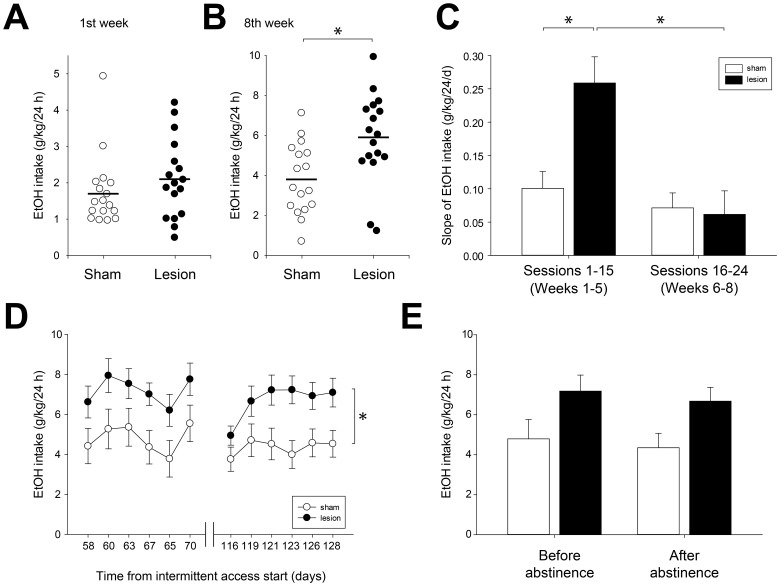
Escalation and stability of voluntary ethanol consumption. (A) Mean ethanol consumption did not differ between sham and lesioned groups in the first week (3 sessions) of IEA. Each symbol indicates the average ethanol intake for a single rat in the first week of IEA. Mean values of each group are indicated by horizontal bars. (B) Lesioned rats drank significantly more ethanol in the eighth week of IEA. (C) Lesioned rats escalated ethanol intake at higher rates than sham rats over the first 5 weeks of IEA (sessions 1–15), but not during the last 3 weeks of access (sessions 16–24). Bars graphs indicate the average slope of ethanol intake over the intervals shown. (D –E). Significant differences in ethanol consumption between groups were stably maintained after a period of abstinence. Rats were withdrawn from IEA for approximately 7 weeks (46 days), and then restored to IEA for an additional two weeks. Lesioned rats drank more than sham rats both before and after this period of abstinence. Asterisk (D) indicates significant main effect of lesion (p<0.05) on ethanol intake.

Average ethanol consumption in the first week did not differ between the two groups, though the lesioned group drank slightly more (Fig 2A; 2.1±0.3 vs. 1.7±0.2 g/kg/24 h in the lesioned and sham groups respectively, NS). By comparison, ethanol intake in the lesioned group was substantially higher than that in the sham group after eight weeks of IEA (Fig. 2B; 5.9±0.5 vs. 3.8±0.4 g/kg/24 h in the lesioned and sham groups respectively, p <0.01). This higher level of intake was achieved by a more rapid escalation of intake in the lesioned group that persisted for approximately the first 5 weeks of ethanol access before intake levels plateaued thereafter (Fig. 1A). Analysis of the slope of increasing ethanol intake in these two intervals (first 5 weeks [sessions 1–15] vs. the last 3 weeks (sessions 16–24) of IEA) showed that the slope was significantly higher in the lesioned group during the first 5 weeks of IEA, but not thereafter (Fig. 2C; significant interaction of lesion x interval, F(1, 32)  = 7.9, p<0.01; p<0.05, posthoc comparing slope of ethanol intake for sham vs. lesioned rats in sessions 1–15). Following the initial period of rapid escalation of ethanol intake, the rate of change in ethanol intake did not differ in lesioned and sham rats (no significant difference between sham and lesioned groups in sessions 16–24, posthoc NS).

To examine the stability of differences in ethanol consumption between the sham and lesioned rats, a subset of rats (n = 7/group) were allowed an initial 10 weeks of IEA, followed by nearly 7 weeks of abstinence (46 days), and then two additional weeks of IEA. Rats were maintained in their home cages during the period of abstinence with *ad lib* food and water supplied as usual. Analysis of 20% ethanol intake showed that lesioned rats consumed more ethanol (Fig. 2D-E; main effect of lesion, F(1, 12)  = 6.5, p<0.05), and that this difference was stably maintained before and after the period of abstinence (sham rats – 4.8±1.0 before and 4.4±0.7 g/kg/24 h after; lesioned rats – 7.2±0.8 before and 6.7±0.7 g/kg/24 h after; no significant effect of time, F(1, 12)  = 3.6, NS; no significant interaction of lesion x time, F(1,12)  = 0.01, NS).

### Taste preference

**Figure 3:**
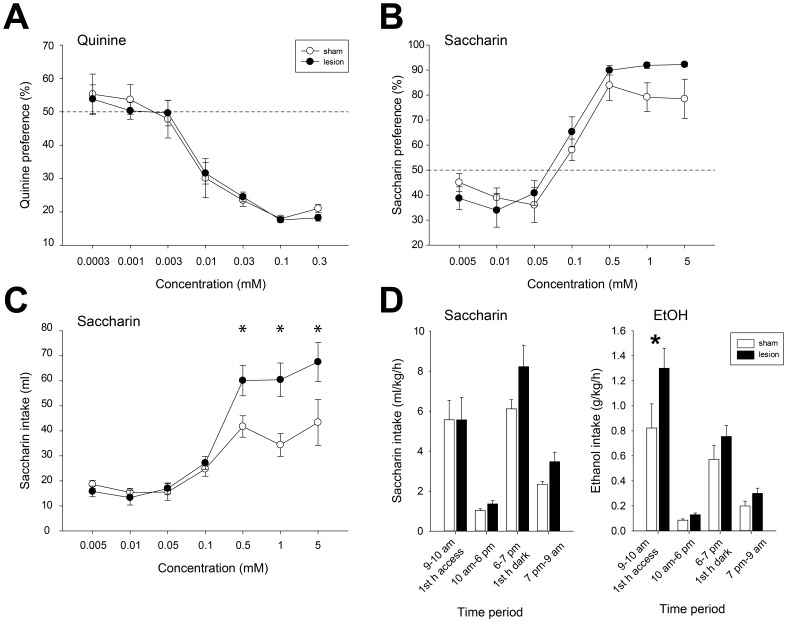
Taste preference. (A) Aversion to quinine did not differ between sham and lesioned rats. (B) Preference for saccharin did not significantly differ between sham and lesioned rats, despite quantitatively increased preference in lesioned rats at saccharin concentrations of 0.5 mM and above. (C) Saccharin intake was significantly higher in lesioned rats at concentrations of 0.5 mM saccharin and above. (D) Timeline of saccharin (left panel) and ethanol (right) intake over 24 hour sessions. The pattern of saccharin intake over 24 hours did not differ between sham and lesioned animals. Rates of intake were similar in the first hour of saccharin access and highest for both groups in the first hour of the dark cycle. By contrast, rates of 20% ethanol intake were higher in lesioned rats specifically during the first hour of access.

Gustatory function contributes to voluntary ethanol intake [35] and habenular lesions have been reported to alter bitter taste aversion [36]. We therefore investigated bitter and sweet taste preference in home-cage two bottle choice experiments in sham and lesioned rats. Quinine preference decreased as a function of concentration in both groups of rats (Fig. 3A; main effect of quinine concentration, F(6, 12)  = 58.7, p<0.001), but did not differ between sham and lesioned rats (no significant effect of lesion, F(1, 12)  = 0.3, NS; no significant interaction of concentration x lesion, F(6, 72)  = 0.3, NS).

Saccharin preference increased as a function of increasing concentration (Fig. 3B; main effect of saccharin concentration, F(6, 12)  = 54.6, p<0.001). There was no significant difference between sham and lesioned groups (no significant effect of lesion, F(1, 12)  = 2.4, NS). Preference for saccharin appeared to be higher at highly preferred saccharin concentrations (0.5 mM and above) but this did not reach significance (no significant interaction of lesion x saccharin concentration, F(6, 72)  = 1.5, NS). Ceiling effects may have obscured differences, however, as preference in both groups was near maximal at concentrations of 0.5 mM saccharin and above. Inspection of the volume of saccharin consumed showed that saccharin intake was significantly higher for lesioned rats at preferred concentrations (Fig. 3C; significant interaction of saccharin concentration x lesion, F(6, 72)  = 5.9, p<0.001). Lesioned rats consumed significantly more than sham rats at saccharin concentrations of 0.5 mM and above (p<0.05, posthoc tests).

To further investigate elevated saccharin and ethanol intake in lesioned animals, we studied the timing of 5 mM saccharin and 20% ethanol ingestion during 24 hour periods of home cage access. The distribution of saccharin intake across the 24 hour cycle was similar for sham and lesioned rats, with both groups consuming saccharin at highest rates during the first hour of the dark cycle (Fig. 3D, left panel; main effect of time, F(1, 12)  = 57.0, p<0.001; drinking rate during 1^st^ hour of dark cycle [6–7 pm] significantly higher than that occurring during all other intervals, p<0.05, posthoc). Though lesioned rats consumed more saccharin than sham rats in every time period except the first hour of access, this difference did not reach statistical significance (no main effect of lesion, F(1, 12)  = 3.2, NS; and no significant interaction of lesion x time, F(3, 36)  = 1.2, NS). By contrast, lesioned rats consumed significantly more ethanol specifically during the first hour of ethanol access (Fig. 3D, right panel; significant interaction of lesion x time, F(3, 36)  = 2.9, p<0.05; lesioned vs. sham intake in 1^st^ hour of access, p<0.05, posthoc). Interestingly, these and previous results [31] suggest that the highest rates of voluntary ethanol intake leading to peak BECs occur within the first hour of access, and suggest that elevated ethanol intake in lesioned rats may be motivated by the pharmacological effects of the drug. Notably, rates of saccharin intake during the first hour of access were nearly identical in sham and lesioned animals.

### Operant self-administration of 20% ethanol

**Figure 4:**
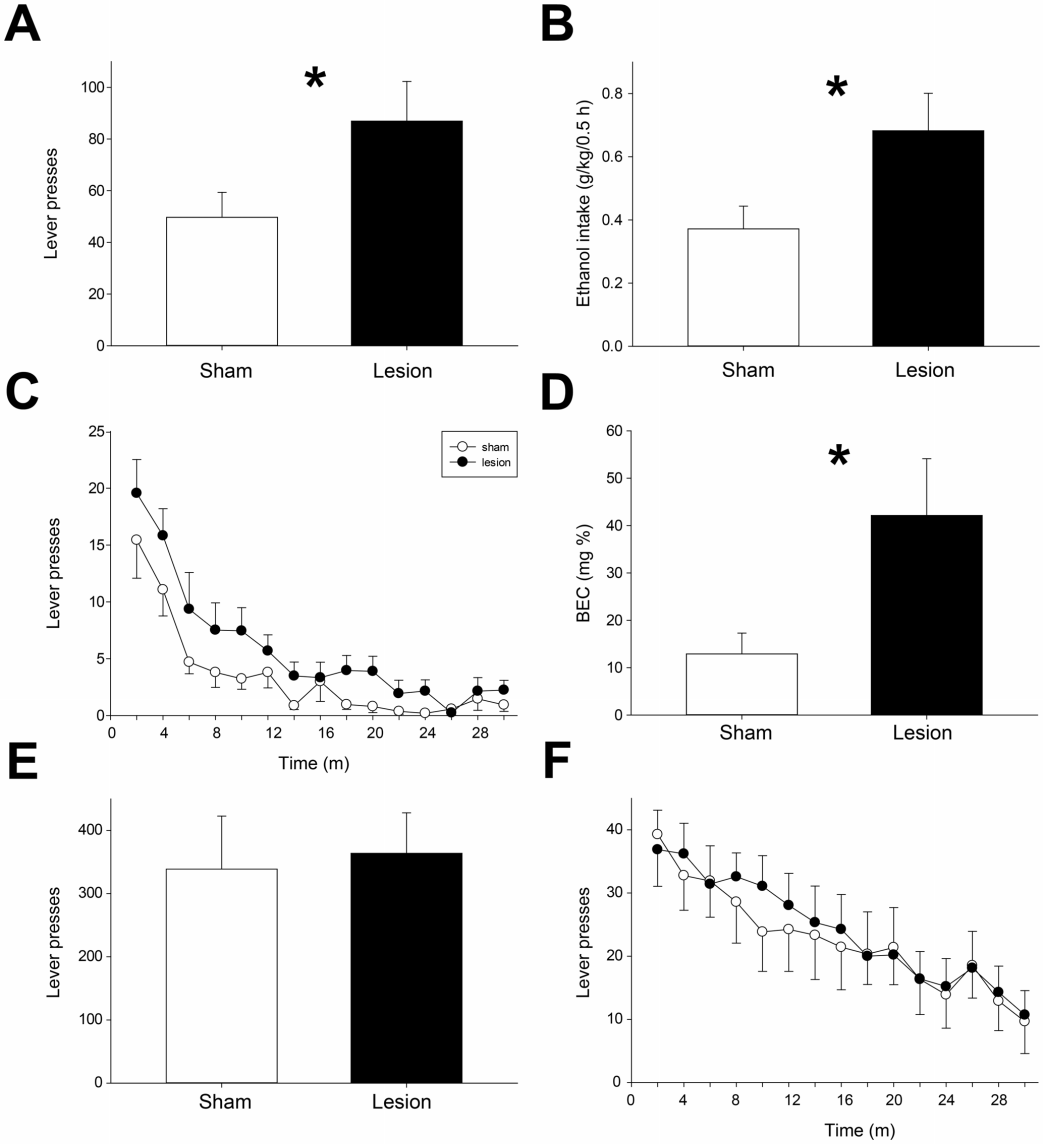
Operant self-administration of ethanol and sucrose. (A-B) Operant responding (A) for 20% ethanol was significantly higher in lesioned vs. sham rats, resulting in elevated levels of ethanol consumption (B). (C) Timing of lever pressing across 30 m self-administration sessions. Compared to sham animals, lesioned rats maintained elevated response rates for the duration of the session. (D) Operant self-administration resulted in significantly higher BECs in lesioned rats compared to sham rats. (E-F) Sham and lesioned rats showed similar levels of operant responding for 2% sucrose (E), and similar patterns of responding over the course of the 30 m session (F).

A total of 19 rats (10 sham and 9 lesioned rats) with a history of IEA were trained to self-administer 20% ethanol on an FR3 schedule. Stable levels of responding (average of last three rewarded sessions) were significantly higher in lesioned vs. sham rats (Fig. 4A; 87±15 vs. 50±10 lever presses for lesioned and sham rats, respectively; t = 2.1, p<0.05). In addition, levels of ethanol intake were higher in lesioned animals (Fig. 4B; 0.68±0.12 vs. 0.37±0.07 g/kg for lesioned and sham rats; t = 2.3, p<0.05). Increased lever pressing in lesioned rats was sustained for the duration of each operant session (Fig. 4C; main effect of lesion, F(1, 17)  = 5.9, p<0.05; main effect of time, F(14, 238)  = 20.5, p<0.001; no significant interaction of lesion x time, F(14, 238)  = 0.6, NS). BECs measured immediately after 30 minute operant response sessions revealed that lesioned rats achieved higher BECs during self-administration sessions (Fig. 4D; 42±12 vs. 13±4 mg/dl for lesioned and sham rats, p<0.05).

### Operant self-administration of 2% sucrose

To determine if increased operant responding in lesioned rats occurred selectively for 20% ethanol, we studied self-administration of 2% sucrose. Sham and lesioned rats reached similar levels of stable operant responding for sucrose (Fig. 4E; 338±84 vs. 363±64 responses per session for sham and lesioned rats, respectively; t = 0.2, NS). In addition, the pattern of operant responding occurring within sessions did not differ between groups (Fig. 4F; significant main effect of time, F(14, 182)  = 29.4, p<0.001; but no significant effect of lesion, F(1, 13)  = 0.4, NS; and no significant interaction of lesion x time, F(14, 182)  = 0.7, NS).

### Extinction and reinstatement of ethanol seeking

**Figure 5:**
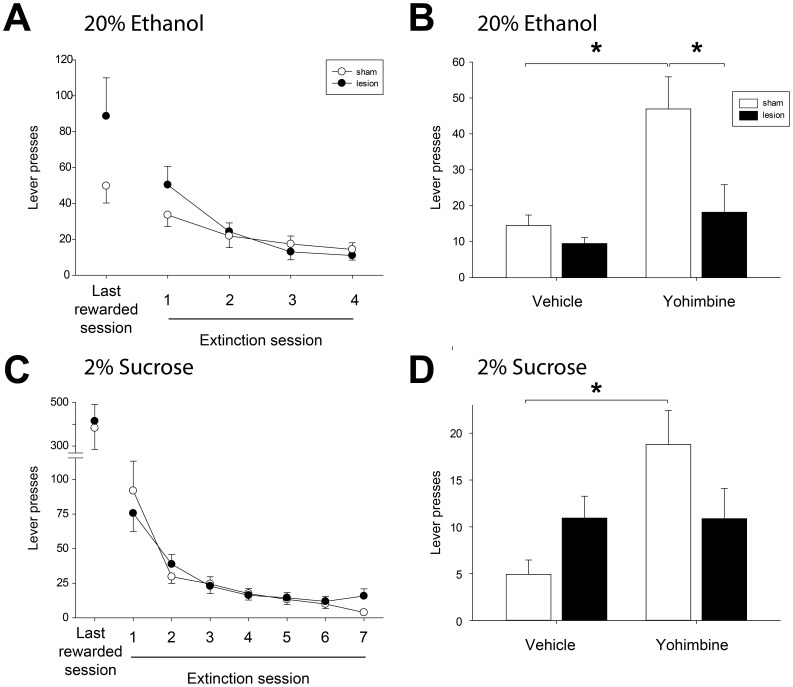
Extinction and reinstatement of ethanol and sucrose seeking. (A) The number of lever presses in lesioned and sham rats did not differ during extinction of operant responding for 20% ethanol. (B) Yohimbine administration reinstated ethanol seeking in sham but not lesioned rats. The number of lever presses by sham rats after yohimbine injection was significantly higher than that occurring after vehicle injection, and higher than lever presses performed by lesioned rats after yohimbine administration. Brackets indicate significant posthoc differences. (C) Sham and lesioned rats showed similar rates of extinction of responding for 2% sucrose. (D) Yohimbine administration induced reinstatement of sucrose seeking in sham but not lesioned rats.

After rats reached stable levels of operant responding for ethanol, self-administration was extinguished over four successive extinction sessions. Lever pressing in the lesioned group was higher than that in sham rats in the first extinction session (Fig. 5A), but there was no significant interaction of lesion x extinction session (F(3, 128)  = 0.7, NS) when ethanol self-administration rates were included as a covariate in the analysis (Fig. 5A, “Last rewarded session”). Mean response rates (± SEM) in the last extinction session were 11±3 lever presses for lesioned rats and 14±4 for sham rats.

Reinstatement of ethanol seeking was tested by administration of yohimbine (2 mg/kg) or vehicle (distilled water) 30 minutes before testing in the extinction paradigm. Vehicle administration resulted in rates of operant responding very similar to those recorded in the final extinction session (Fig. 5B; 10±2 vs. 15±3 lever presses in lesioned and sham rats, respectively). Yohimbine caused a robust increase in operant responding in sham rats, but had no significant effect on responding in lesioned animals (18±8 vs. 47±9 lever presses in lesioned and sham rats, respectively; significant interaction of lesion x drug F(1, 17)  = 4.8, p<0.05). Post hoc analyses showed that yohimbine administration increased lever pressing in sham rats relative to the lesioned group and relative to vehicle administration in the sham group (p<0.05, posthoc tests). Thus, lesion of the LHb blocked the ability of yohimbine to reinstate ethanol seeking.

Responses on the inactive lever did not differ between groups, nor were they affected by drug administration (Table 3; no effect of lesion, F(1, 17)  = 1.2, NS; no effect of drug, F(1, 17)  = 0.8, NS; no interaction of lesion x drug, F(1, 17)  = 3.6, NS).

**Table 3:** Inactive lever presses following vehicle or yohimbine administration during reinstatement of ethanol or sucrose seeking.

	Group	Vehicle	Yohimbine
20% Ethanol	Sham	1.2±0.5	0.8±0.2
	Lesioned	0.9±0.4	2.1±0.7
2% Sucrose	Sham	1.1±0.4	1.3±0.4
	Lesioned	1.7±0.6	1.8±1.2

### Extinction and reinstatement of 2% sucrose seeking

To determine if reinstatement deficits in lesioned animals were specific for ethanol seeking, we studied yohimbine-induced reinstatement of sucrose seeking in ethanol-naïve rats. After animals reached stable levels of 2% sucrose self-administration, responding was extinguished over seven extinction sessions. Lever press responses declined rapidly across extinction sessions (Fig. 5C; main effect of extinction day, F(6, 78)  = 34.7, p<0.001) in both sham and lesioned rats (no effect of lesion, F(1, 13)  = 0.4, NS; no interaction of lesion x day, F(6, 78)  = 1.0, NS). Mean response rates in the final extinction session were 16±5 lever presses for lesioned rats and 4±1 for sham rats.

Similar to the results seen for reinstatement of ethanol seeking, yohimbine reinstated sucrose seeking in sham but not lesioned rats (Fig. 5D; significant interaction of lesion x drug, F(1, 13)  = 6.5, p<0.05; also main effect of drug, F(1, 13)  = 6.4, p<0.05). Yohimbine administration resulted in a significant increase in operant responding in the sham group relative to operant responding after vehicle administration (19±4 vs. 5±2 lever presses after yohimbine and vehicle, respectively; p<0.05, posthoc). A trend toward increased operant responding in sham vs. lesioned rats was apparent after yohimbine administration (p = 0.06, posthoc test). Yohimbine did not reinstate sucrose seeking in the lesioned group (11±3 vs. 11±2 lever presses after yohimbine and vehicle, respectively; NS). Inactive lever presses did not differ between sham and lesioned rats, nor were they affected by drug administration (Table 3; no effect of lesion, F(1, 13)  = 0.2, NS; no effect of drug, F(1, 13)  = 0.0, NS; no interaction of lesion x drug, F(1, 13)  = 0.2, NS).

### Conditioned taste aversion

**Figure 6:**
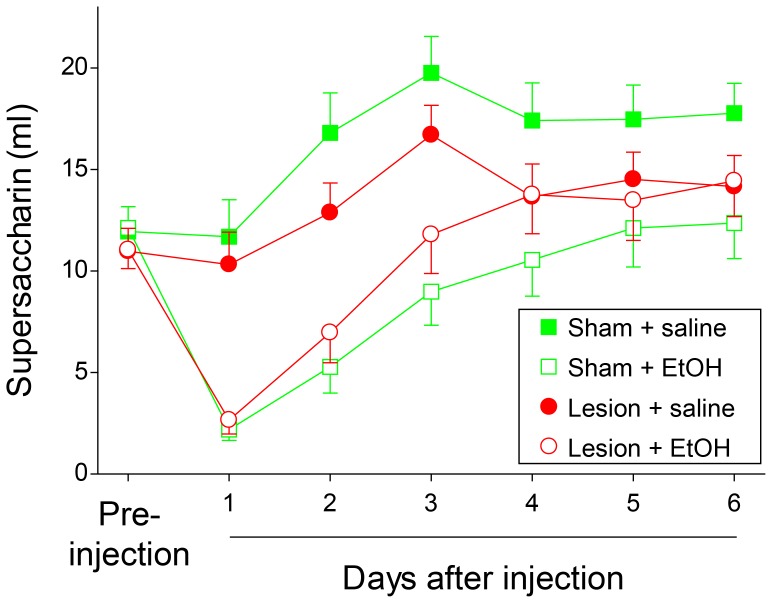
Ethanol induced conditioned taste aversion. LHb lesion attenuated the magnitude of a taste aversion conditioned by a single injection of ethanol. X-axis shows intake of supersaccharin; y-axis shows time of saccharin intake relative to injection in days. Squares and circles indicate sham- and LHb-lesioned rats, respectively. Filled and open symbols indicate saline and ethanol injection groups, respectively.

To determine if LHb lesion affected learning caused by ethanol’s aversive effects, we examined ethanol-induced CTA in lesioned and sham rats. Rats in each group were injected with saline or ethanol (0.7 g/kg, IP) after an initial 30 minute period of supersaccharin access in the home cage. Ethanol administration conditioned an aversion to supersaccharin in both sham and lesioned groups as demonstrated by reduced supersaccharin intake after ethanol injection (Fig. 6; main effect of drug, F(1, 73)  = 40.9, p< 0.001; also main effect of time, F(3.2, 238.0)  = 7.2, p<0.001). However, the magnitude of this aversion was dependent on surgical treatment (significant interaction of drug x lesion, F(1, 73)  = 6.6, p<0.05). Posthoc testing revealed that ethanol-induced CTA was attenuated in lesioned rats relative to sham animals, as supersaccharin consumption was significantly higher in lesioned vs. sham rats after ethanol injection (10.7±0.9 vs 7.7±1.0 ml, respectively; p<0.05). Lesioned and sham rats consumed similar amounts of supersaccharin on the first day after ethanol injection (Fig. 6, day 1), but lesioned rats appeared to recover from this initial aversion more rapidly than sham operated rats (consumption on days 2–6). However, there was no significant time-dependent difference in drug effects on sham vs. lesioned groups (no significant drug x lesion x time interaction; F(3.2, 238.0)  = 1.0, NS). In lesioned and sham rats that received control saline injections, lesioned animals consistently consumed less supersaccharin than sham animals (days 1–6), but this did not reach statistical significance.

### Histological confirmation of lesions

**Figure 7:**
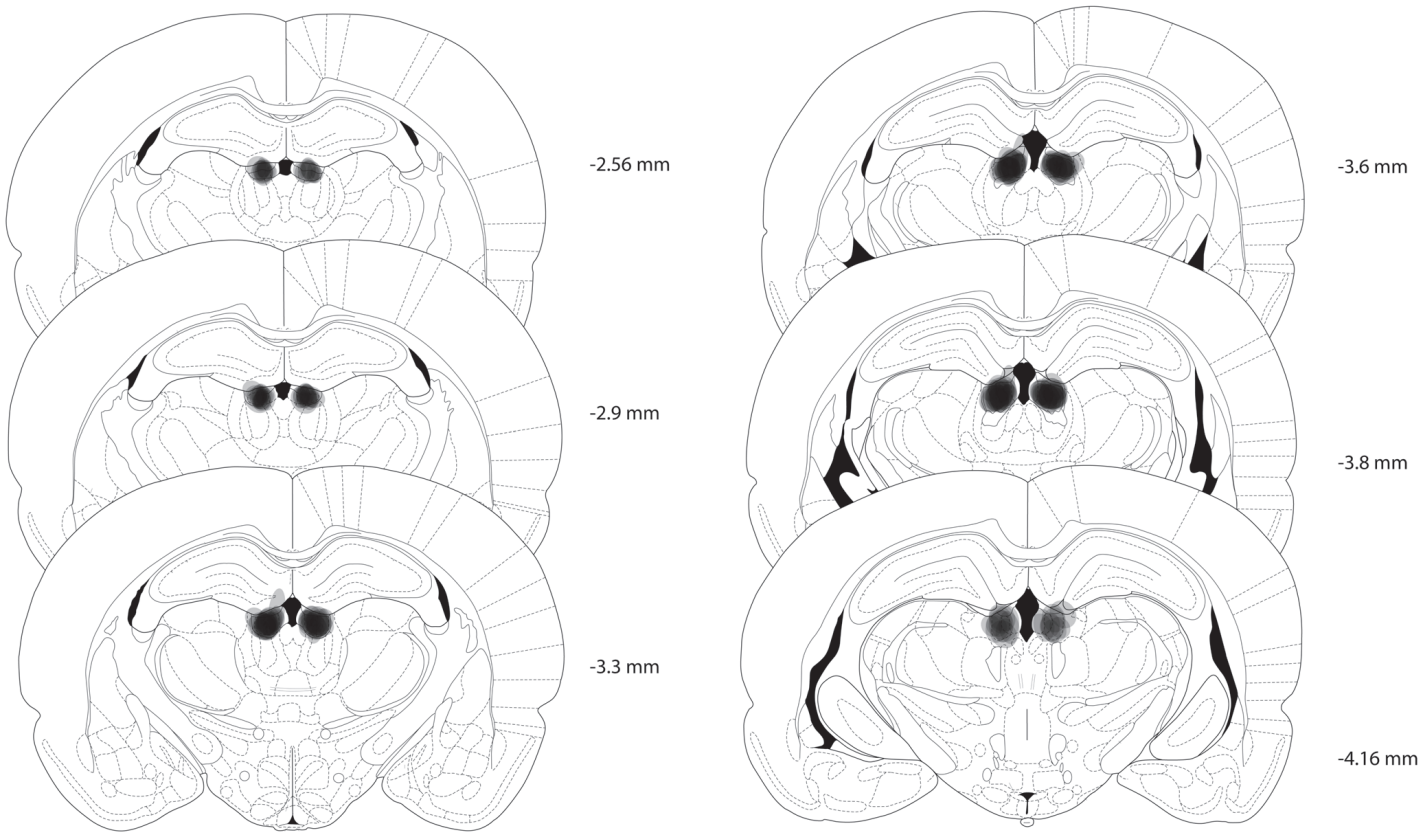
Lateral habenula lesions. Lesion sites were centered on the lateral habenula. Semi-transparent shading shows lesion sites overlaid for each rat, so that darkest areas indicate areas of greatest damage. In some rats, lesions extended to nearby structures (particularly including the medial habenula, medial thalamic areas, and the dentate gyrus). Numbers to the right of each section indicate anterioposterior position relative to bregma.

Lesions were largely confined to the LHb (Figure 7), though they encroached upon neighboring structures, including the medial habenula (MHb). To determine if lesions of the MHb contributed to increased ethanol intake in lesioned animals, we quantified damage to this structure in lesioned rats tested in the IEA paradigm. Damage to the MHb ranged from a maximum of 50% (complete lesion of the MHb in one hemisphere) to 0%, and averaged 20±5%. Voluntary ethanol intake during IEA did not differ in rats with low vs. high MHb damage (5±2% vs. 33±5% damage, respectively, and ethanol intake of 6.3±0.4 and 5.8±0.9 g/kg/24 h in the last week of IEA in low and high damage groups, respectively; no effect of MHb damage, F(1, 13)  = 0.5, NS; and no interaction of MHb damage x drinking session, F(23, 292)  = 0.7, NS), suggesting that damage to this structure did not contribute to the results reported here.

## Discussion

Our results show that the LHb plays an important role in controlling ethanol-directed behaviors. LHb lesion increased the rate at which rats escalated voluntary ethanol consumption in a two bottle choice paradigm, resulted in higher levels of maintained intake relative to sham rats both before and after a period of abstinence, and gave rise to higher BECs relative to sham animals. Operant self-administration of ethanol was also increased in lesioned rats, again resulting in higher BECs than those measured in sham animals. In addition, yohimbine-induced reinstatement of ethanol and sucrose seeking were blocked in animals with LHb lesions. Finally, our results show that ethanol CTA was significantly attenuated by LHb lesion. These results demonstrate an important role for the LHb in regulating ethanol-seeking and consumption and moreover show that LHb circuits contribute to learning driven by ethanol’s aversive effects.

### Effects of LHb lesion on ethanol intake and self-administration

LHb lesions did not acutely increase voluntary ethanol consumption, but rather increased the rate at which escalation of intake occurred, leading to higher sustained levels of ethanol consumption. Ethanol intake in the first week of the IEA paradigm was similar in sham and lesioned groups (Figs. 1A and 2A). Drinking levels in the two groups diverged gradually, with LHb-lesioned rats escalating their intake more rapidly than those in the sham group (Figs. 1A and 2C). Drinking in lesioned rats plateaued around the 6^th^ week of IEA, at which time ethanol intake in lesioned rats was approximately 50% higher than that in the sham group. This difference in intake was maintained for the remainder of the IEA paradigm.

Changes in taste preference are unlikely to contribute to increased ethanol intake and self-administration in LHb-lesioned rats. Though lesions of the habenular complex have been reported to decrease quinine intake [36], [37], we found that sham and lesioned rats showed nearly identical dose-dependent aversion to quinine (Fig. 3A). LHb lesion clearly increased consumption of preferred saccharin concentrations (Fig. 3C; concentrations of 0.5 mM and higher). However, patterns of elevated saccharin and ethanol intake differed, suggesting distinct physiological mechanisms underlying consumption of the two solutions (Fig. 3D). Saccharin consumption occurred at highest rates during the first hour of the dark cycle (6-7 pm). In contrast, rates of ethanol intake were highest for both groups during the first hour of access (9-10 am), and were significantly higher in the lesioned group exclusively during this period. Because drinking during this interval is likely to result in the highest BECs [31], this result suggests that increased ethanol intake in lesioned rats during this interval may be motivated by the pharmacological effects of the drug.

Our findings of increased ethanol intake and self-administration in LHb-lesioned animals contribute to a growing body of evidence implicating the habenula in regulating drug seeking behaviors. Habenular circuits have been shown to play a central role in regulating learning driven by negative outcomes [6]–[11], and recent studies have shown that this function importantly extends to regulation of drug-seeking behaviors [6], [14], [15]. Electrical stimulation of the LHb accelerates extinction of cocaine self-administration and attenuates subsequent cocaine-induced reinstatement [15], consistent with a mechanism in which excitation of LHb neurons suppresses drug-seeking through aversive conditioning. Jhou and colleagues [6] have shown that cocaine induces delayed excitation in a subset of LHb and downstream RMTg neurons, and that optogenetic inhibition of RMTg firing specifically during this period of delayed excitation blocks cocaine-conditioned avoidance behaviors. In addition, studies of mechanisms regulating nicotine intake suggest an analogous role for circuits originating in the medial habenula in mediating aversion-dependent suppression of nicotine self-administration [14].

Increased ethanol intake and self-administration in LHb-lesioned animals may also arise through deficits in learning from aversive drug outcomes. Ethanol causes dose-dependent aversive effects that include nausea, sedation, motoric impairment and hangover effects [16]. These effects are thought to condition a learned aversion to ethanol that acts to decrease subsequent intake, as suggested by the inverse correlation of ethanol CTA and voluntary ethanol intake [18], [19]. Our results show that lesion of the LHb causes attenuation of a taste aversion conditioned by a single noncontingent ethanol injection. LHb neurons are known to be excited by negative stimuli, including drug-induced negative stimuli [1], [2], [6]. Loss of an ethanol-induced aversive signal after LHb ablation could thus contribute to increased ethanol intake in lesioned rats. In this regard, our finding that LHb lesion did not acutely increase voluntary ethanol consumption, but rather increased the rate at which escalation of intake occurred, is suggestive of an impairment in ethanol-induced aversion learning. However, additional experiments are needed to determine if attenuated CTA in LHb-lesioned animals plays a causal role in increasing voluntary ethanol intake.

### Effects of LHb lesion on extinction and reinstatement of ethanol and sucrose seeking

Given evidence of an important role for the LHb in aversive conditioning, lesion of this structure might be expected to impair extinction learning, particularly as lesion and stimulation of the LHb respectively impair and accelerate extinction of cocaine-seeking [15], [38]. However, we found no extinction deficits in lesioned rats. Our results agree with those reported in a recent study of cocaine self-administration, in which operant responding in an initial extinction test was unaltered by acute pharmacological inactivation of the LHb [39]. These findings contrast with those of a recent study showing that LHb lesions block extinction learning in rats trained to self-administer cocaine [15]. Methodological differences (bilateral electrolytic lesions vs. unilateral excitotoxic lesion) and/or the drug tested could contribute to these divergent results. It is also possible that recovery of neural function may have contributed to intact extinction learning in our lesioned animals, as operant responding in extinction was tested weeks after lesions were made.

The effects of LHb lesion were not specific to reinstatement of ethanol seeking, as yohimbine-induced reinstatement of sucrose seeking was also blocked in lesioned animals. Our results extend recent findings that pharmacological inactivation of the LHb blocks yohimbine-induced potentiation of cue-dependent reinstatement of cocaine-seeking, as well as attenuating yohimbine-induced anxiety-related behaviors [39]. Habenular neurons, particularly those in the medial subdivision of the LHb, are activated by an array of stressful stimuli, including foot shock, novel environments, restraint stress, food restriction and lithium chloride injection [29], [30], [40], [41]. Habenula lesions impair stress-induced potentiation of prepulse inhibition, decrease avoidance learning tested under high stress conditions, and block the development of learned helplessness in response to inescapable shock [42]–[44]. Taken together, these results demonstrate an extensive role for habenular function in mediating stress-induced behavioral responses. These results may have clinical implications, as they raise the possibility that individual differences in LHb function could importantly contribute to vulnerability to stress-induced relapse of drug seeking in abstinent alcoholics.

In summary, our study provides novel evidence of an important role for the LHb in a number of ethanol directed behaviors. Our results show that LHb lesions increase voluntary ethanol intake and operant-self administration and block yohimbine-induced reinstatement of ethanol seeking. Further, LHb lesions attenuate ethanol CTA. These results are likely to have important implications for mechanisms underlying alcohol use disorders as studies in human volunteers show that vulnerability to developing an alcohol use disorder is inversely related to sensitivity to acute ethanol effects, including the aversive effects of the drug [21]–[23].
