# Correction: Lesions of the Lateral Habenula Increase Voluntary Ethanol Consumption and Operant Self-Administration, Block Yohimbine-Induced Reinstatement of Ethanol Seeking, and Attenuate Ethanol-Induced Conditioned Taste Aversion

**DOI:** 10.1371/journal.pone.0098732

**Published:** 2014-05-23

**Authors:** The PLOS ONE Staff

“Brain Institute” is incorrectly listed as an author for this article. The correct author list for this article is:

Andrew K. Haack, Chandni Sheth, Andrea L. Schwager, Michael S. Sinclair, Shashank Tandon, Sharif A. Taha
